# Similarities and Differences in Chinese and Caucasian Adults' Use of Facial Cues for Trustworthiness Judgments

**DOI:** 10.1371/journal.pone.0034859

**Published:** 2012-04-13

**Authors:** Fen Xu, Dingcheng Wu, Rie Toriyama, Fengling Ma, Shoji Itakura, Kang Lee

**Affiliations:** 1 Department of Psychology, Zhejiang Sci-Tech University, Hangzhou, China; 2 State Key Laboratory of Cognitive Neuroscience and Learning, Beijing Normal University, Beijing, China; 3 Dr. Eric Jackman Institute of Child Study, University of Toronto, Toronto, Canada; 4 Department of Psychology, Kyoto University, Kyoto, Japan; 5 Department of Psychology, University of California San Diego, San Diego, California, United States of America; University of Cambridge, United Kingdom

## Abstract

**Background:**

All cultural groups in the world place paramount value on interpersonal trust. Existing research suggests that although accurate judgments of another's trustworthiness require extensive interactions with the person, we often make trustworthiness judgments based on facial cues on the first encounter. However, little is known about what facial cues are used for such judgments and what the bases are on which individuals make their trustworthiness judgments.

**Methodology/Principal Findings:**

In the present study, we tested the hypothesis that individuals may use facial attractiveness cues as a “shortcut” for judging another's trustworthiness due to the lack of other more informative and in-depth information about trustworthiness. Using data-driven statistical models of 3D Caucasian faces, we compared facial cues used for judging the trustworthiness of Caucasian faces by Caucasian participants who were highly experienced with Caucasian faces, and the facial cues used by Chinese participants who were unfamiliar with Caucasian faces. We found that Chinese and Caucasian participants used similar facial cues to judge trustworthiness. Also, both Chinese and Caucasian participants used almost identical facial cues for judging trustworthiness and attractiveness.

**Conclusions/Significance:**

The results suggest that without opportunities to interact with another person extensively, we use the less racially specific and more universal attractiveness cues as a “shortcut” for trustworthiness judgments.

## Introduction

Trust is fundamental to interpersonal relationships. Thus, all cultures in the world place paramount value on interpersonal trust. Typically, interpersonal trust is developed gradually over time based upon the outcomes of many social interactions between the trustor and the trustee [1]. With time, we learn about whether a person is trustworthy by finding out the four key elements that make up our judgments of another's trustworthiness [2]: ability (Is the person capable?), benevolence (Is the person nice?), honesty (Is the person truthful?), and reliability (Is the person reliable?)

However, in many social situations, we may not be afforded the luxury of time to find out about a person's trustworthiness in such depth. Fast trust decisions have to be made based on limited information. It has been suggested that the most critical time frame for establishing a trust relation is at the beginning of the two parties' first encounter or interaction [3]. This is perhaps why first impressions play such an important role in our social lives (e.g., evaluation of political candidates [4]–[5]). Among the many cues that individuals use to form the first trusting impressions, are those on the face [6]. Willis et al. [7] even found that a novel face can convey the information about a person's trustworthiness within 100 ms!

Recently, researchers have begun to speculate that certain facial properties may specifically contain information about a person's trustworthiness [8]. Todorov et al. [9] were the first to examine this question empirically. They asked participants to rate the trustworthiness of novel faces generated by Facegen, a computer program that can systematically manipulate specific facial features (e.g., the size of the eyes or mouth). They found that brow ridge (down/up), cheekbones (shallow/pronounced), chin (wide/thin), and nose sellion (shallow/deep) were correlated significantly with participants' trustworthiness judgments of the faces. Furthermore, the first three features could be used to reliably predict participants' trustworthiness judgments of novel faces, suggesting that individuals consistently use certain facial cues for trustworthiness judgments.

Interestingly, trustworthiness has been consistently found to be highly correlated with attractiveness judgments: The more attractive a face is judged, the more trustworthy it is deemed [6]–[7], [10]. However, it is entirely unclear whether and to what extent the facial features for trustworthiness found by Todorov et al. [9] are also being used for judging attractiveness. Here, we tested the hypothesis that individuals use the same facial attractiveness features for judging facial trustworthiness as a “shortcut”. This hypothesis is based on the idea that during the initial encounter with another person, because we have not had sufficient amount of interaction with the person, we do not have sufficient time to learn about the person's ability, honesty, benevolence, and reliability to make a more informed decision about the person's trustworthiness. To circumvent this problem, we may use facial attractiveness as a heuristic or “shortcut” because the cues for facial attractiveness are both readily accessible and useful indicators of biological and social dispositions. Indeed, extensive evidence has shown that attractive individuals not only are healthier and more likable than unattractive individuals [11]–[14], but also tend to have and be deemed to have positive personality traits [14]–[20], including honesty [15] that is an important component of trustworthiness [2].

To test this attractiveness heuristic hypothesis, we examined the facial cues used for judging Caucasian faces' trustworthiness and attractiveness by Caucasian participants who were naturally highly familiar with Caucasian faces and by Chinese participants who had no direct contact with Caucasian individuals. The reasons we asked Caucasian and Chinese participants to rate the trustworthiness of Caucasian faces were two-fold. First, all of the existing studies of facial trustworthiness mainly asked Caucasian participants to judge the trustworthiness of Caucasian faces. The involvement of Caucasian participants in the present study allowed for the assessment of whether our procedures could replicate the existing findings regarding the relationship between facial attractiveness and trustworthiness. Second, because Chinese participants had no direct contact with Caucasian individuals, if Chinese participants used similar strategies as Caucasian participants to assess Caucasians' trustworthiness (i.e., using certain facial features) and if their judgments were to be closely linked to their facial attractiveness judgments, it would provide strong evidence to support the face attractiveness heuristic hypothesis: Individuals, as a shortcut, would use the more universal and less experience dependent facial attractiveness cues for trustworthiness.

Existing evidence is in fact equivocal regarding the validity of the attractiveness heuristic hypothesis. On one hand, it has been shown that due to the important role of attractiveness in evolutionary adaptation, there is a strong universal component to facial attractiveness that does not vary by race nor by experience. Individuals from different races tend to have consensus regarding whether certain individuals are attractive or unattractive [21]. Given the high correlation between facial attractiveness and trustworthiness found in the previous studies [6]–[7], [10], it is possible that it may be a universal strategy for us to use the attractive cues for judging the trustworthiness of faces regardless of whether we have any experience with the type of to-be-judged faces. If that is the case, Chinese participants would not only use attractiveness cues for judging the trustworthiness of Caucasian faces but would also use similar cues as Caucasians for making such judgments.

On the other hand, the existing studies about facial cues for social judgments have so far only asked participants to judge trustworthiness of own-race faces [9], [22]–[24]. Extensive studies that have examined the processing of own- and other-race faces have mainly focused on the recognition and categorization of such faces. They have consistently found that we process own- and other-race faces differently due to the lack of experience with other-race faces [25]–[26]. In addition, it has been suggested that experience should influence perceptions of facial attractiveness despite the fact that it has a strong universal component [27]. Further, Stanley et al. [28] recently demonstrated that race plays an important role in both trustworthiness judgments and trust behaviors, although they did not examine facial cues to trustworthiness per se. These existing findings seem to suggest that Chinese and Caucasian participants may differ significantly in their use of facial cues for trustworthiness judgments. However, no evidence exists to confirm or disconfirm this suggestion, which was tested in the present study.

In the present study, we recruited Caucasian participants to judge the trustworthiness of Caucasian faces and Chinese participants who had no direct interaction with Caucasian individuals to judge the same faces. We used a data-driven statistical model of 3D faces to generate emotionally neutral faces which could be adjusted on 61 shape features and 36 texture features. All of the feature controls are linear transformations of independent components, which are used to represent every single face in the data-driven model constructed from the Principle Component Analysis [29]. Participants were asked to rate trustworthiness and attractiveness of the generated faces. We examined whether and to what extent the same set of facial features contribute to participants' judgments of facial attractiveness and trustworthiness, and whether and to what extent Chinese and Caucasian participants use the same set of facial cues for making such judgments.

## Methods

### Participants

Seventy-six Caucasian young adults (37 males, *M* age = 22.4 years, range = 18.4–34.0 years) and 68 Chinese young adults (33 males, *M* age = 20.5 years, range = 17.3–28.6 years) participated. They had normal or corrected-to-normal vision. All procedures were approved by the local ethics committees (Institutional Review Board (IRB) of the State Key Laboratory of Cognitive Neuroscience and Learning of Beijing Normal University; The Research Ethics Policy and Advisory Committee (REPAC) of the University of Toronto). Participants gave written informed consent before the experiment and they were compensated for their participation. Caucasian participants were recruited in Canada. Only Chinese participants who had no direct interaction with Caucasian individuals were recruited in China.

### Materials

**Figure 1:**
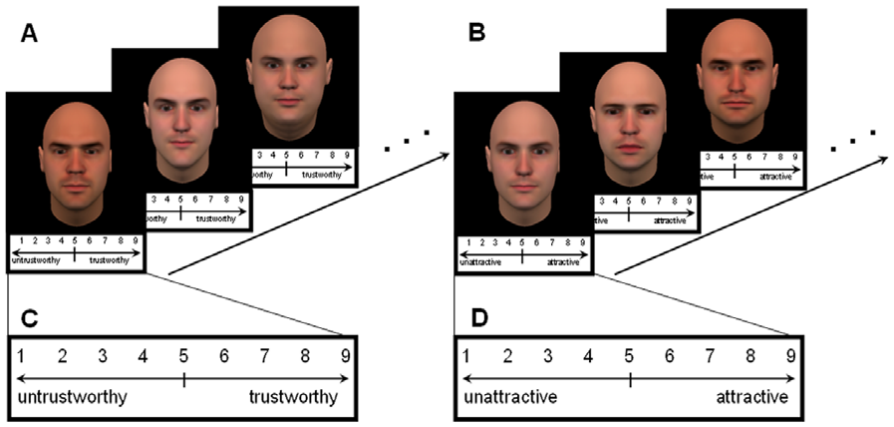
The procedure of face judgments. A: the procedure of trustworthiness judgments; B: the procedure of attractiveness judgments; C: scale used in the trustworthiness judgments; D: scale used in the attractiveness judgments. In both tasks, the faces were presented with the scale underneath the face image.

Facegen Modeller 3.1 (http://facegen.com) was used to generate emotionally neutral faces with direct gazes for the present study. The face models used in Facegen are based on 3D laser scans of faces. The 3D faces created by Facegen Modeller 3.1 can be adjusted in 61 shape features and 36 texture features. Three hundred Caucasian male faces were generated randomly with the following adjustments. First, race of faces was set to European (Caucasian faces) using Facegen's race controls, because faces created completely randomly by Facegen could be of any race. Second, because all faces created by Facegen are without any hair, we adjusted all of them to male faces using Facegen's gender controls. Third, since the participants in the present study were around 20 years old, the age of faces was set between 20 to 30 years using Facegen's age controls such that the participants were asked to evaluate faces within the age range to which they were currently mostly exposed. Fourth, to avoid influences of symmetry on trustworthiness judgments, all faces were set to be symmetrical. These procedures resulted in 300 bitmap face images with a resolution of 400×400 pixels (see Fig. 1 for an example).

### Procedure

Participants were seated in a comfortable chair in a quiet room facing a computer screen, in which faces were presented in the center. First, participants were asked to judge three blocks of faces in terms of trustworthiness based on their first impression. The 300 faces were presented individually in a randomized order in every block. Underneath each face, a nine-point scale was presented in which 1 represented extremely untrustworthy and 9 represented extremely trustworthy with 5 in the middle (Fig. 1). Participants were asked to press the corresponding number on the keyboard to rate the faces. Each face was presented until the participants responded upon which the next face was immediately presented. Also, in an additional single block, participants were also asked to rate the attractiveness of each of the 300 faces on the same 9 point scale with 1 representing extremely unattractive and 9 representing extremely attractive (Fig. 1).

## Results

### Trustworthiness judgments by Caucasian and Chinese Participants

The original ratings of 1 to 9 were linear-transformed to −4 to 4. Therefore, negative ratings represented untrustworthy or unattractive judgments, positive numbers represented trustworthy or attractive judgments, and 0 represented judgments in the middle. First, we confirmed that the trustworthiness judgments were highly reliable in both Chinese (Cronbach α = 0.98) and Caucasian (Cronbach α = 0.99) participants, and attractiveness judgments were also highly reliable in both Chinese (Cronbach α = 0.97) and Caucasian (Cronbach α = 0.97) participants.

**Figure 2:**
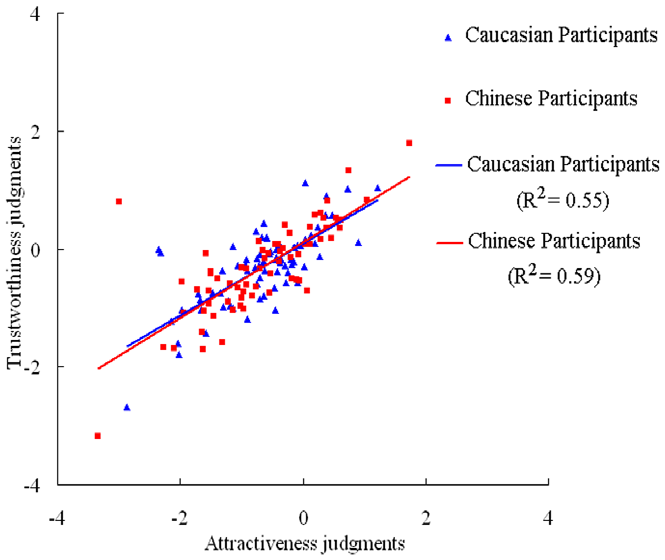
The correlations between attractiveness and trustworthiness judgments of Caucasian male faces by Caucasian and Chinese participants.

Then, we averaged the ratings of all faces to obtain the mean overall ratings of trustworthiness or attractiveness for every participant. One-way ANOVA showed that there were no differences between the Caucasian and Chinese participants in both the trustworthiness [*F* (1, 142) = 0.07, *p*>0.1; Chinese (−0.32) vs. Caucasian (−0.29)] and the attractiveness judgments [*F* (1, 142) = 0.04, *p*>0.1; Chinese (−0.67) vs. Caucasian (−0.64)]. Paired T-tests showed that the trustworthiness and the attractiveness judgments are significantly different from each other by both Caucasian [*t* (75) = 5.76, *p*<0.001] and Chinese [*t* (67) = 4.67, *p*<0.01]. However, the two evaluations are highly correlated with each other for both races of participants (Pearson Correlation, Caucasian: *R^2^* = 0.55, *p*<0.001, *N* = 76; Chinese: *R^2^* = 0.59, *p*<0.001, *N* = 68) (Fig. 2), which replicated the results of previous studies.

### Facial cues of trustworthiness

To assess the relative predictive power of facial cues on trustworthiness of faces, we performed two stepwise regression analyses, where all possible models were processed with high probability of F-to-enter (*p* = 0.001) and F-to-remove (*p* = 0.1) to avoid multicollinearity problems. Variance inflation factors (VIF) were used to delete variables with potential multicollinearity problems (VIF>2 [30]) For the first regression analysis, we assigned each face a trustworthiness score, which was the mean trustworthiness rating by Caucasian participants for the particular face. These trustworthiness scores were used as the predicted variable, and all of the 61 shape features and the 36 texture features were used as the predictors. For the second analysis, we assigned each face the mean trustworthiness rating by Chinese participants as the predicted variable, and all of the 61 shape features and the 36 texture features were used as the predictors. The final accepted models based on the ratings by Caucasian and Chinese participants were both significant [Caucasian: *R^2^* = 0.71, *F*(10, 289) = 70.23, *p*<0.001; Chinese: *R^2^* = 0.72, *F*(10, 289) = 72.41, *p*<0.001]. Inspection of the models suggested that facial cues for trustworthiness by Chinese and Caucasian participants were highly similar (Table 1).

**Table 1:** Facial features that significantly contributed to the trustworthiness judgments of Caucasian male faces by Caucasian and Chinese.

	By Caucasian		By Chinese		
	Part correlations	Standardized coefficient	Part correlations	Standardized coefficient	Higher Trustworthiness
**Skin Shade - dark/light**	−0.40	−0.41	−0.52	−0.54	darker
**Brow Ridge Inner - down/up**	0.37	0.39	0.21	0.23	more up
**Cheekbones - shallow/pronounced**	0.11	0.12	0.15	0.17	more pronounced
**Face - heavy/light**	0.29	0.30	0.25	0.25	lighter
**Forehead - tall/short**	−0.30	−0.34	−0.25	−0.27	taller
**Face - brow-nose-chin ratio**	−0.28	−0.30	−0.27	−0.29	smaller
**Forehead - small/large**	−0.22	−0.22	−0.14	−0.14	smaller
**Eyes - small/large**	0.14	0.15	0.13	0.14	larger
Nose – short/long	−0.08	−0.09	–	–	shorter
Mouth - Lips deflated/inflated	0.14	0.15	–	–	more inflated
Mouth - drawn/pursed	–	–	−0.13	−0.15	more drawn
Head - thin/wide	–	–	0.16	0.17	wider

*p*<0.001; bolded features are significant for both Chinese and Caucasian participants.

**Figure 3:**
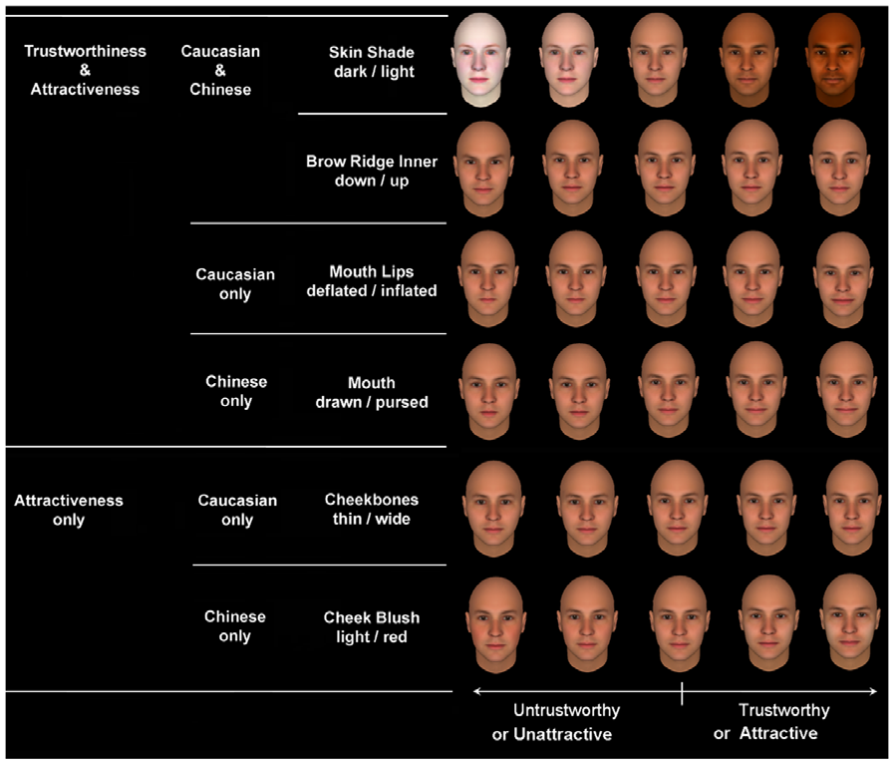
Examples of facial cues for trustworthiness and attractiveness judgments by Caucasian and Chinese participants. These facial features are adjusted in terms of −4, −2, 0, 2, and 4 SD from the mean.

Eight facial features were significantly uniquely correlated with the trustworthiness judgments of Caucasian male faces by both Chinese and Caucasian participants (see the bolded features in Table 1). For example, faces with darker skin shades were rated as more trustworthy by both Chinese and Caucasian participants (Fig. 3). Table 1 also showed that there were several unique significant trust cues used by either Chinese or Caucasian participants, respectively. Only two facial features were used by Caucasian but not Chinese participants. Similarly, only two facial features were used by Chinese but not Caucasian participants.

### Facial cues of attractiveness

To test the hypothesis that we use the same facial features for judging facial trustworthiness and attractiveness, the predictive power of facial cues on attractiveness of faces were assessed. The same regression analyses as those for trustworthiness were performed on all of the 61 shape features and the 36 texture features were used as the predictors and the attractiveness ratings were used as the predicted variable. The models for Caucasian and Chinese data were both significant [Caucasian: *R^2^* = 0.65, *F*(10, 289) = 54.66, *p*<0.001; Chinese: *R^2^* = 0.64, *F*(11, 288) = 46.66, *p*<0.001]. Inspection of the models suggested that facial cues for attractiveness by Chinese and Caucasian participants were also very similar to each other (Table 2).

**Table 2:** Facial features that significantly contributed to the attractiveness judgments of Caucasian male faces by Caucasian and Chinese.

	By Caucasian		By Chinese		
	Part correlations	Standardized coefficient	Part correlations	Standardized coefficient	Higher Attractiveness
**Skin Shade - dark/light**	−0.44	−0.46	−0.45	−0.47	darker
**Face - heavy/light**	0.40	0.41	0.30	0.31	lighter
**Cheekbones - shallow/pronounced**	0.11	0.13	0.13	0.16	more pronounced
**Forehead - tall/short**	−0.22	−0.24	−0.22	−0.25	taller
**Brow Ridge Inner - down/up**	0.14	0.15	0.15	0.16	more up
**Face - brow-nose-chin ratio**	−0.15	−0.15	−0.25	−0.27	smaller
Cheekbones - thin/wide	−0.14	−0.15	–	–	thinner
Mouth - wide/thin	−0.09	−0.11	–	–	wider
Chin - pronounced/recessed	−0.13	−0.14	–	–	more pronounced
Mouth - lips deflated/inflated	0.12	0.15	–	–	more inflated
Forehead - small/large	–	–	−0.12	−0.12	smaller
Eyes - small/large	–	–	0.14	0.15	larger
Mouth - drawn/pursed	–	–	−0.15	−0.17	more drawn
Head - thin/wide	–	–	0.12	0.13	wider
Cheek Blush - light/red	–	–	−0.12	−0.13	lighter

*p*<0.001; bolded features are significant for both Caucasian and Chinese participants.

**Figure 4:**
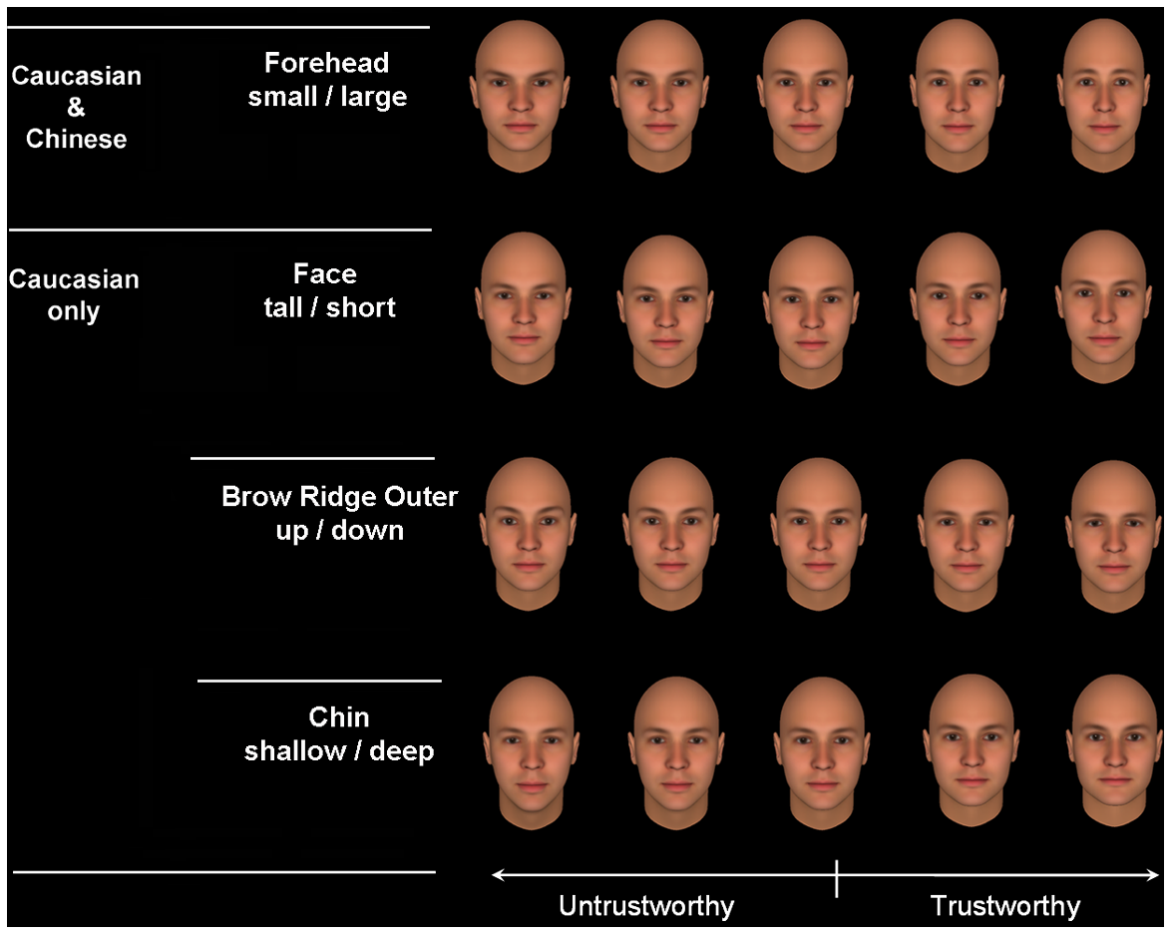
Examples of facial cues for trustworthiness with attractiveness considered by Caucasian and Chinese participants. These facial features are adjusted in terms of −4, −2, 0, 2, and 4 SD from the mean. Note that after controlling attractiveness, the facial cues of trustworthiness for Chinese only concluded skin shade (dark/light) which is shown in Fig. 3.

Six facial features were significantly and uniquely correlated with the attractiveness judgments of Caucasian male faces by both Chinese and Caucasian participants (see the bolded features in Table 2). For example, as shown in Figure 4, faces with darker skin shades were rated as more attractive by both Chinese and Caucasian participants. Table 2 also showed several uniquely significant features used by either Chinese or Caucasian participants, respectively, to judge the attractiveness of the faces. Interestingly, there were many more different facial cues between Caucasian and Chinese for attractiveness than those for trustworthiness. Specifically, four facial features were used by Caucasian but not by Chinese participants, and five facial features were used by Chinese but not Caucasian participants.

### Comparisons between facial cues for trustworthiness and attractiveness

Direct comparisons showed that most of the cues used in judging trustworthiness of Caucasian male faces were also used in judging attractiveness, especially by Chinese participants (Table 3, see Fig. 3 for examples). It should be noted that most of the same cues for trustworthiness and attractiveness were used by both Caucasian and Chinese participants. Only one facial cue for both judgments was used by Caucasian participants only, whereas four facial cues for both judgments were used by Chinese participants only. The results of the comparisons also showed that there existed limited facial cues for attractiveness but not for trustworthiness in Caucasian participants, and there were limited facial cues for attractiveness but not for trustworthiness in Chinese participants. Interestingly, forehead (small/large) and eyes (small/large) were cues for both trustworthiness and attractiveness for Chinese participants, but it was only a cue for trustworthiness for Caucasian participants.

**Table 3:** Comparisons of facial cues between trustworthiness and attractiveness.

	Caucasian	Chinese
***Cues for both trustworthiness and attractiveness***		
Skin Shade - dark/light	√	√
Brow Ridge Inner - down/up	√	√
Cheekbones - shallow/pronounced	√	√
Face - heavy/light	√	√
Forehead - tall/short	√	√
Face - brow-nose-chin ratio	√	√
Mouth - lips deflated/inflated	√	
Forehead - small/large		√
Eyes - small/large		√
Head - thin/wide		√
Mouth - drawn/pursed		√
***Cues for attractiveness only***		
Cheekbones - thin/wide	√	
Chin - pronounced/recessed	√	
Mouth - wide/thin	√	
Cheek Blush - light/red		√
***Cues for trustworthiness only***		
Forehead - small/large	√	
Eyes - small/large	√	
Nose – short/long	√	

### Facial cues of trustworthiness with attractiveness partialled out

The above results from the direct comparisons between cues for trustworthiness and attractiveness showed that there were few cues specific to trustworthiness judgments relative to attractiveness. To ascertain whether the two types of judgments were indeed statistically similar, we re-ran the two regression analyses of trustworthiness but this time we used a hierarchical regression model where the attractiveness factor was entered in the first block, followed by the facial features in the second block. For the attractiveness factor, we used the mean attractiveness rating of each face by either Caucasian or Chinese participants. For the regression analysis involving the data from Caucasian participants, the first model that included only the mean attractiveness rating was significant δ*R^2^* = 0.79, δ*F*(1, 298) = 1104.63, *p*<0.001]. Also, the second model that included the factors of facial features was significant, [δ*R^2^* = 0.18, δ*F*(95, 203) = 13.40, *p*<0.001]. For the data from Chinese participants, the first model involving only attractiveness was significant [δ*R^2^* = 0.93, δ*F*(1, 298) = 3757.25, *p*<0.001]. The second model involving facial features was also significant [δ*R^2^* = 0.05, δ*F*(95, 203) = 4.58, *p*<0.001]. Thus, although facial attractiveness accounted for most of the variance of trustworthiness ratings, after partialling out the effect of attractiveness, participants (Caucasian participants in particular) still used some unique facial features for trustworthiness judgments (Table 4, see Fig. 4 for examples). In other words, these facial features are specific trust cues above and beyond those for attractiveness.

**Table 4:** Facial features significantly contributed to the trustworthiness judgments of Caucasian male faces by Caucasian and Chinese with attractiveness considered.

	By Caucasian		By Chinese		
	Part correlations	Standardized coefficient	Part correlations	Standardized coefficient	Higher Trustworthiness
**Forehead - small/large**	−0.11	−0.29	−0.04	−0.11	smaller
Face - tall/short	−0.04	−1.86	–	–	taller
Face - up/down	0.07	1.65	–	–	more down
Face - brow-nose-chin ratio	−0.06	−0.27	–	–	smaller
Mouth - wide/thin	0.06	0.96	–	–	thinner
Chin - shallow/deep	0.05	0.25	–	–	deeper
Brow ridge outer - up/down	0.05	0.56	–	–	more down
Chin - wide/thin	0.05	0.32	–	–	thinner
Skin shade - dark/light	–	–	−0.07	−0.14	darker

*p*<0.001; bolded features are significant for both Caucasian and Chinese participants.

As can be seen in Table 4, Caucasian participants had more cues that uniquely accounted for a significant amount of variance for trustworthiness ratings in comparison to Chinese participants. For Caucasian participants, most trustworthy cues were not used for attractiveness judgments. In contrast, for Chinese participants, there were only two cues that uniquely accounted for a significant amount of variance in trustworthiness ratings, but these two cues were also accounted for a significant amount of variance in attractiveness judgments.

## Discussion

Previous studies suggested that an unfamiliar person's trustworthiness can be gleaned by some specific facial features [9], [22]. Using a model-based approach [6], [31], the present study tested the hypothesis that without extensive interactions with others, we use facial attractiveness cues for judging trustworthiness as a shortcut. To do so, we systematically examined the similarities and differences of facial cues in Caucasian and Chinese participants' judgments of trustworthiness and attractiveness of Caucasian male faces. We first examined the facial cues for trustworthiness judgments used by Chinese and Caucasian participants and those for attractiveness judgments to compare the similarities or differences between the two types of judgments. Then, we partialled out the effect of attractiveness to assess whether and to what extent there were specific cues for trustworthiness but not attractiveness by the two groups of participants.

Our regression analyses showed that attractiveness accounted for a large amount of the variance in participants' judgments of facial trustworthiness. These findings along with the significant correlations between trustworthiness and attractiveness as revealed in this study and in previous studies [6]–[7], [10], suggest that when individuals form the first impression of another's trustworthiness, they largely rely on facial attractiveness. These findings are in line with those from several neuroimaging and neuropsychological studies that show trustworthiness judgments of faces to be as spontaneous and automatic as attractiveness judgments [8]–[9], [32]–[34]. Also, Willis and Todorov [7] revealed that only trustworthiness judgments were as fast as attractiveness judgments among trait judgments such as aggressiveness and likability. Thus, the present and existing findings taken together suggest that relying on facial attractiveness for trustworthiness judgments could be an efficient strategy when we encounter unfamiliar individuals.

Unlike the previous studies, we compared the facial cues for attractiveness and trustworthiness by participants who were either highly experienced or inexperienced with the type of faces that they had to judge. Regardless of their experiences, the Caucasian and Chinese participants used almost identical facial features for judging trustworthiness. The major significant cues that were used by the two groups of participants included such facial features as skin shade (the darker, the more trustworthy), brow ridge inner (the more up, the more trustworthy), cheekbones (the more pronounced, the more trustworthy), face heaviness (the lighter, the more trustworthy), forehead (the taller and smaller, the more trustworthy), and the face's brow-nose-chin ratio (the smaller, the more trustworthy).

It should be noted that for both Caucasian and Chinese participants, the direction of the relationship between the facial features (i.e., whether a facial feature is strong or weak) and ***trustworthiness*** (i.e., whether a face is deemed trustworthy or not) was also identical. Further, these facial features were also major significant cues used by Caucasian and Chinese participants for judging ***attractiveness***. Again, the direction of the relationship between these features and facial attractiveness was the same for the two groups of participants. Thus, these findings support the hypothesis that participants used the more universal and non-race-specific facial attractiveness cues for judging the trustworthiness of unfamiliar individuals. Further support for this hypothesis came from our additional regression analyses. When we added the attractiveness to the regression model as a regressor, it accounted for 79% of the variance in Caucasian participants' trustworthiness judgments and 93% of the variance in Chinese participants' trustworthiness judgments. These findings taken together strongly support the attractiveness heuristic hypothesis: That without opportunities to interact with another person extensively, we use the less racially specific and more universal attractiveness cues as a ‘shortcut’ for trustworthiness judgments.

However, once the effect of attractiveness factor was partialled out, cross-race differences emerged. There were only two facial cues that still uniquely accounted for a significant amount of variance in Chinese participants' judgments of trustworthiness. These two features were also significant features for facial attractiveness judgments used by Chinese participants. This finding suggests that Chinese participants without any direct contact with Caucasian individuals used attractiveness facial cues for judging trustworthiness of Caucasian faces, providing further support to our attractiveness heuristic hypothesis. In contrast, Caucasian participants seemed to use some unique facial cues that appeared specific for facial trustworthiness but not for attractiveness judgments. They were face - tall/short, face - up/down, mouth - wide/thin, chin - shallow/deep, brow ridge outer - up/down, and chin - wide/thin. It should be noted that these facial cues independently accounted for very limited amount of variance of trustworthiness (see part correlations presented in Table 4). The reason could be the high correlations between these features and other features in the Facegen model (see Fig. 4). For Caucasian but not for Chinese, these specific facial cues are working together with other significant features (e.g., size of forehead and height of brow ridge inner in Fig. 4).

The differences between Caucasian and Chinese participants in the specific facial cues for trustworthiness might be a result of their differential experience with Caucasian faces. Specifically, for Caucasians, a large amount of exposure to their own race faces allows them to distinguish slight differences between trustworthy and untrustworthy faces. This might be the reason that they not only use the universal and non-race-specific attractiveness cues, but also use unique cues above and beyond the contribution of the attractiveness factor. More direct empirical evidence is needed to ascertain why these cues were specific to Caucasian participants. One possibility is the socialization of trustworthiness cues. For example, in comic books, theatrical plays, and movies in the West, the main heroes may be typically depicted or played by actors with these unique facial features. Another possibility is that these facial cues could be related to baby-faceness. Baby-faceness was shown to be different between East Asian and Caucasian in its relationship with personality traits such as sociability [14]. In addition, Oosterhof and Todorov (2008) showed that trustworthiness and sociability were highly correlated with each other [6]. Thus, we speculate that baby-faceness should be positively correlated with trustworthiness, and the correlation could be different between different races, which might have led to the different trustworthiness facial cues used by Caucasian and Chinese participants in the present study.

Further, it should be noted that the present study only asked Caucasian and Chinese participants to judge Caucasian faces, and the faces they judged were synthetic and artificial. Using real faces, Zebrowitz et al (1993) [15] also found that faces with larger eyes were perceived as more attractive by East Asian participants (i.e., Korean) but not by Caucasian. However, they did not find any overlap in terms of attractiveness cues between East Asian participants and Caucasian participants, which is different from the present study. One possible reason for this difference could be that the facial cues they examined were much more limited than we did here.Further studies across more races and using real faces are needed to ascertain facial cues of trustworthiness for specific races and generalize the present findings.

The present findings add to the recently growing body of evidence that suggest the face to be an important source for obtaining shallow but immediate information about another's personal traits when in-depth information is not available due to time and resource constraints. For example, in addition to attractiveness and trustworthiness, researchers have identified the facial width-to-height ratio in Caucasian males to be related to their perceived aggression [22], [31], [35] and this judgment has recently been shown to be independent of experience [36]. In addition to aggression, studies have shown that individuals could readily use faces to make judgments about a host of personal traits such as competence, dominance, and warmth [5], [37]–[38].

These judgments have been shown to have significant predictive powers. For example, the United States' congressional election outcomes could be predicted based on the judgments of competences shown on the candidates' faces [5], judgments of dominance could predict the final rank obtained by West Point cadets [37], judgments of power and warmth based on the faces of the CEOs of Fortune 500 companies could predict their company's financial success [38], and trustworthiness ratings of faces were more accurate when the faces belonged to truly trustworthy individuals than untrustworthy individuals [39]. Further, existing studies even showed that specific personal traits and behaviors could be predicted by some facial cues. For example, facial width-to-height ratio could be used to predict trust behaviors [24] and unethical behaviours [40] in men, and actual aggression levels as measured by penalty minutes [35]. In addition, to move this growing field of facial psychomorphology forward, one must not only be contented with establishing the linkages between perceived personal traits based on the face and the personal traits themselves. Rather, we must, through empirical research, identify exactly what facial cues are indicative of personal traits as illustrated by this study and those by McCormick and her associates [23]. Furthermore, we must also identify the underlying biological mechanisms that are responsible for the trait-specific facial morphology. Only with use of a rigorous and comprehensive approach, can we avoid the pitfalls that brought the demise of phrenology, and empirically establish the linkages between face morphology, biology, and personality psychology, and thus the scientific facial psychomorphology.
